# Acute Toxicity Assessment and Prediction Models of Four Heavy Metals

**DOI:** 10.3390/toxics11040346

**Published:** 2023-04-06

**Authors:** Xin Zheng, Chao Wei, Juntao Fan, Xinyu Liu, Yin Hou, Jianan Ling, Jian Wei, Peiyuan Liu

**Affiliations:** 1 State Key Laboratory of Environmental Criteria and Risk Assessment, Chinese Research Academy of Environmental Sciences, Beijing 100012, China; 2 School of Life Sciences, Tianjin University, Tianjin 300072, China

**Keywords:** water quality criteria, native species, species sensitivity distribution, warm-water fish, toxicity data

## Abstract

Water quality criteria (WQC) are developed to protect aquatic organisms. Toxicity data of local fish are essential to improve the applicability of WQC derivatives. However, the paucity of local cold-water fish toxicity data limits the development of WQC in China. *Brachymystax lenok* is a representative Chinese-endemic cold-water fish, which plays an important role in the characterization of metal toxicity in the water environment. Whereas, the ecotoxicological effects of copper, zinc, lead and cadmium, as well as its potential as a test species for the metal WQC, remain to be investigated. In our study, acute toxicity tests of copper, zinc, lead and cadmium were performed on this fish according to the OECD method and 96 h-LC_50_ values were calculated. The results showed that the 96 h-LC_50_ values of Cu^2+^, Zn^2+^, Pb^2+^ and Cd^2+^ for *B. lenok* were 134, 222, 514 and 734 μg/L, respectively. Toxicity data for freshwater species and Chinese-native species were collected and screened, and the mean acute values of each metal for each species were ranked. The results showed that the accumulation probability of zinc by *B. lenok* was the lowest and less than 15%. Thus, *B. lenok* was sensitive to Zn and can be considered as the test cold-water fish for derivation of Zn WQC. In addition, *B. lenok* in comparison with warm-water fish, we found that cold-water fish are not always more sensitive to heavy metals than warm-water fish. Finally, the models for toxic effects prediction of different heavy metals on the same species were constructed and evaluated the reliability of the model. We suggest that the alternative toxicity data provided by the simulations can be used to derive WQC for metals.

## 1. Introduction

Copper (Cu^2+^), zinc (Zn^2+^), lead (Pb^2+^) and cadmium (Cd^2+^) are the common heavy metals contaminants in the environment [1,2,3]. In conjunction with rapid economic development and progressive urbanization, heavy metal pollution in aquatic ecosystems has increased. Recently, severe heavy metal pollution has become widespread in Chinese rivers and lakes [4,5]. Many studies have investigated the toxic effect of heavy metals on various aquatic organisms and compared their sensitivity. For example, tests of acute and chronic toxicity have shown that the mottled sculpin is more sensitive to Cd, Cu and Zn than the rainbow trout [6]. A study of acute toxicity in four different hydra species showed that Cu was more toxic than Cd and that Cd was more toxic than Zn [7]. In addition, the combined toxicities of several heavy-metal pollutant mixtures to *Daphnia* were compared with the toxicities of single pollutants; species sensitivities based on LC_50_ were then evaluated [8].

Similarly, in a study comparing copper sensitivity differences between aquatic species in China and the United States based on an species sensitivity distributions (SSDs) method, the sensitivity of different species to heavy metals in different regions is also different. In China, non-arthropods are more sensitive to copper than arthropods, and the reverse is true in the United States. Chinese fishes, arthropods, and vertebrates were more sensitive than those in the United States [9]. In general, fish are less sensitive to metals than arthropods and non-arthropods [9].

Water quality criteria (WQC) are threshold limits for pollutants developed to ensure appropriate protection for more than 95% of organisms from adverse effects [10,11]. Recently, China has issued the technical guideline for deriving WQC for freshwater organisms [12] and developed national environmental criteria [12,13]. Species sensitivity distributions (SSDs) are generally used to derive WQC [14]. SSD curves are constructed based on toxicity data and cumulative probabilities for individual species [15]. WQC are derived from the HC_5_ (hazardous concentration) of the SSD, which protects 95% of all species from adverse effects [16]. The US EPA recommends that toxicity data from at least three phyla and eight families be used to construct SSDs [17]. In China, toxicity data from at least three phyla and ten species are required for SSD construction, including at least one cyprinid fish and one non-cyprinid fish [18].

Several studies have investigated the effects of heavy metals on standard laboratory fish, such as zebrafish (*Danio rerio*) and fathead minnows [18,19], which provides a strong technical support for the development of WQC in China. In addition, studies have explored the sensitivity of many fish not native to China using data from international databases such as ECOTOX. However, little is known about the sensitivity of many fish species endemic to China to metal contaminants. In addition, as Chinese WQC have primarily been extrapolated based on data from non-native fish species, it is uncertain whether native fish species are adequately protected. Therefore, it is particularly necessary to assess the sensitivity of native species in relation to metal contaminants, in order to adequately protect Chinese aquatic ecosystems.

In previous studies, cold-water fish were more sensitive to metal contaminants than warm-water fish. e.g., sculpins [20] and sturgeons [6]. However, the response of common warm-water fish, such as Cyprinids, which are native to China, to various metal contaminants has been well studied [21]. Due to the relatively few toxicity studies on cold-water fish, it is unknown whether other cold-water fish are more sensitive to metal pollutants than warm-water fish [22]. *Brachymystax lenok* is a cold-water fish in the Salmonidae [23], which is perfect for our work. This ancient species is found in eastern Siberia, Mongolia, China and South Korea. *B. lenok* is widely distributed in northeastern China, primarily Heilongjiang, the Yalu River, the Tumen River and the upper Liao River. This indigenous fish has a high nutritional and economic value, but it is threatened due to overexploitation and pollution. As *B. lenok* has been successfully cultured in an artificial hatchery [24] and is thus suitable as a toxicological test species, limited data are available regarding the effects of acute exposure to typical heavy metals on this species. Therefore, in the present work, *B. lenok* was chosen to test acute toxicity of Cu, Zn, Pb and Cr.

Toxicity tests of aquatic organisms have been widely used as a traditional and convenient method for evaluation of pollutants [25]. Meanwhile, another method for addressing the uncertainty in species sensitivity is the development of models. Herein, both toxicity experiments and model prediction are important approaches for obtaining toxicity data. In summary, the objectives were to: (1) perform acute toxicity study from Cu, Zn, Pb and Cd on *B. lenok*, and 96h-LC_50_ was obtained; acute toxicity data were sequenced with freshwater species and local Chinese species to compare metal susceptibility of *B. lenok* with other species. The applicability of *B. lenok* can be evaluated as the test species. Additionally, another objective was (2) to see if cold water fish are more sensitive than warm water fish. Finally, another objective was to (3) establish a model for predicting the toxic effects of different heavy metals on the same species and evaluate the reliability of the model. Provide basic toxicity data for WQC derivation.

## 2. Materials and Methods

### 2.1. Test Organism

Some energetic and healthy *B. lenok* (~45 days post-hatching) were obtained from the Qinglong Coldwater Breeding Base at the Jilin Fisheries Research Institute (Changchun, China). All selected fish were normally developed, free of any apparent malformations, and of similar size (mean length: 2.31 ± 0.12 cm; mean weight: 72 ± 3 mg). The *B. lenok* were washed with 0.1% (*w*/*v*) NaCl solution and acclimated in a feeding tank for one week before experimentation. The *B. lenok* were exposed to a 13-h light/11-h dark cycle and fed 1.5–2.0% of body weight at 18 ± 1 °C. The death rate was lower than 5% during domestication, which met requirements of OECD Guideline No. 203 [26] and GB/T 27861-2011 [27]. Ten fish were introduced into each test solution and the control solution.

### 2.2. Experimental Design

The endpoint of toxicitydata include LC_50_, EC_50_, LOEC, NOEC, MTAC etc., which are usually selected according to the characteristics of aquatic organisms. For example, EC_50_ is commonly used to calculate the toxic endpoint of algae, NOEC is often used to calculate the toxic endpoint of Daphnia, and LC_50_ is often used to calculate the toxic endpoint of fish. The median lethal concentration (LC_50_) is the concentration of pollutants that cause 50% of individual deaths in a group of tested fish. This is also a standardized method to assess the toxicity.

The acute tests of *B. lenok* to Cd(NO_3_)_2_, Zn(NO_3_)_2_, Cu(NO_3_)_2_, or Pb(NO_3_)_2_ were performed following OECD chemical testing guidelines [23]. The above reagent specifications are analytically pure and are derived from Shanghai Luyou Chemical Technology Co., Ltd. (Shanghai, China). Tap water, which had been thoroughly aerated for >48 h prior to use, was used for all dilutions. For each heavy metal, we developed five serial solutions, increasing concentration by a factor of 1.6 in each solution. Thus, we tested 100, 160, 250, 400 and 630 µg/L Cu and Zn and 400, 630, 1000, 1600 and 2560 µg/L Pb and Cd. The water was changed every 24 h. Exposure concentrations were analyzed by inductively coupled plasma-mass spectrometry (ICP-MS; Agilent 7500a, Santa Clara, CA, USA). The standard solution of four metals is 100 µg/mL. The nebulizer is a concentric nebulizer, and the spray chamber is a small volume swirl spray chamber with semiconductor refrigeration device. The temperature of the cooling circulating water device is 15–20 °C. The main operating parameters of the ICP-MS were as follows: the radio-frequency power was 1480 W; the radio-frequency voltage was 1.85 V; the carrier gas flow was 1.16 L/min; and the sampling depth was 6.0 mm. The limits of detection for Cu^2+^, Zn^2+^, Pb^2+^ and Cd^2+^ were 0.08, 0.67, 0.09 and 0.05 µg/L, respectively. During the acute toxicity tests, some parameters of the tap water were tested. The temperature of the water used in the tests was 17–20 °C, and the measured pH levels in the test solutions were 7.29 ± 0.25 (Table S1). The dissolved oxygen concentrations were 9.36 ± 0.26 mg/L (>80% of air saturation) in all test solution (Table S1). Water hardness was 106 mg/L (calculated by CaCO_3_), within a range of 90–110 mg/L. Ten fish were tested at each experimental concentration and in the control. At the end of the test, the mortality in the control solution did not exceed 10%, as recommended by the OECD guidelines [25].

Under the conditions described above, each fish was exposed to one of several aqueous solutions, where each solution contained a different concentration of heavy metals. The concentration range for each heavy metal was established after preliminary experiments (Table S2). Acute toxicity tests were conducted using serial solutions with geometrically-increasing (by a factor of 1.6) heavy metal concentrations: 100, 160, 250, 400 and 630 µg/L for Cu(NO_3_)_2_ and Zn(NO_3_)_2_ and 400, 630, 1000, 1600 and 2560 µg/L for Pb (NO_3_)_2_ and Cd(NO_3_)_2_. Exposure time was 96 h. Fish mortality in each solution was recorded at 24, 48, 72 and 96 h to determine the LC_50_. The 96 h-LC_50_ was calculated using the probit method [26,28].

### 2.3. Collection of Toxicity Data

Toxicity data for the four heavy metals tested were obtained from the ECOTOX database and from the literature. Only acute test endpoints for aquatic animals were selected, including the 48 h-LC_50_ or -EC_50_ for *Daphnia* or midge *B. lenok* and the 96 h-LC_50_ or -EC_50_ for fish, mollusks, shrimp, and other organisms. Data originating from studies using unsuitable exposure times, untested dilution water, unscientific experimental designs, and relatively insensitive life stages were excluded. When multiple toxicity data were available for a given species, the geometric mean acute values (SMAVs) of the data were calculated and ranked. Then, organisms were ranked based on the SMAV value for each heavy metal (from least to most); this ranking reflected the sensitivity level of each organism. The cumulative probability of species was defined as (the order of the data point)/(1 + total number of data points). Species with lower cumulative probabilities were more sensitive to heavy metals.

### 2.4. Model Development and Verification

Each pair of metals (containing three or more common species in both at least) were used to develop the linear regression models. The relationship between sensitivity of the surrogate and predicted metals are described as:

Log_10_ (*Predicted Toxicity*) = a × Log_10_ (*Surrogate Toxicity*) + b (1)

Here, *Predicted Toxicity* represents the LC_50_/EC_50_ value of the projected metal, *Surrogate Toxicity* is the LC_50_/EC_50_ value of the substitute metal, a is the slope of the regression line, and b represents the intercept.

After removing the models with insignificant correlation (*p*-value > 0.05), the remaining models are verified internally and externally. The leave-one-out cross-validation method is used for internal verification. External validation of the model was performed using measured toxicity data obtained in this study. Root-mean-square error of prediction (RMSEP) between the actual value and the prediction value are used to measure the accuracy of the model.

## 3. Results

### 3.1. Acute Toxicity of B. lenok Exposed to Heavy Metals

Acute toxicity tests were performed using *B. lenok* according to the OECD method, and the exposure concentration gradients for each heavy metal were showed in Table 1. The result showed that *B. lenok* mortality rate increased with increased heavy metal concentrations. Based on the dose-effect relationship, the 96 h-LC_50_ value for each metal was calculated using the probit method. The 96 h-LC_50_ values of Cu^2+^, Zn^2+^, Pb^2+^ and Cd^2+^ for *B. lenok* were 134, 222, 514 and 734 μg/L, respectively (Table 1). The 96 h-LC_50_ of Cd was approximately six times greater than the 96 h-LC_50_ of Cu. As heavy metals with lower 96 h-LC_50_ were more toxic to *B. lenok* and *B. lenok* was most sensitive to Cu, followed by Zn, Pb and Cd.

### 3.2. The Sensitivity of B. lenok among the Freshwater Species

**Figure 1:**
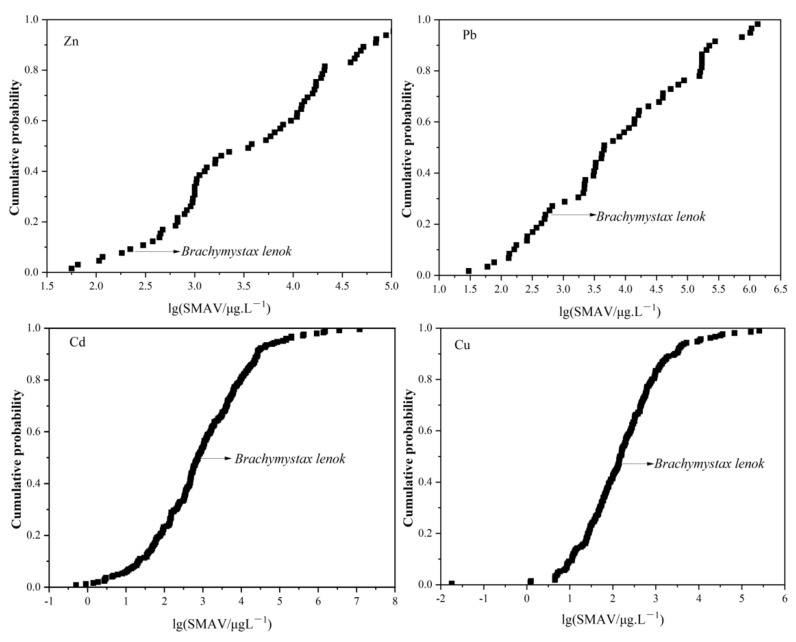
Species sensitivity distributions for the four heavy metals, across all available species.

The acute toxicity data for the four heavy metals were gathered according to the principles for screening data and were shown on Tables S3–S6. SMAVs were subsequently calculated and ranked to compare the sensitivity of *B. lenok* to other species (Figure 1 and Table 2). We obtained toxicity data for Cu, Zn, Pb and Cd in 209, 64, 58 and 245 species, respectively. Within those 209, 64, 58 and 245 species, the rank of *B. lenok* was 98, 6, 14 and 122, respectively. The cumulative probabilities of Cu, Zn, Pb and Cd, calculated as (the order of the data point)/(1 + total number of data points), were 46.67%, 9.23%, 23.73% and 49.59%, respectively.

Thus, the cumulative probability of *B. lenok* for Cu and Cd, with respect to the other species assessed, was approximately 50%, indicating that *B. lenok* is not particularly sensitive to Cu and Cd. The cumulative probability of *B. lenok* for Zn, with respect to the other species assessed, was less than 15%. Generally, it is recommended that sensitive species be used to derive WQC [29]. The cumulative probabilities of sensitive species are lower than the cumulative probabilities of non-sensitive species [30]. A previous study suggested that species with cumulative probabilities less than 15% were suitable recommended as test species [31,32]. Thus, we recommend *B. lenok* as a suitable test species for the development of Zn WQC.

### 3.3. The Sensitivity of B. lenok among the Native Freshwater Species

**Figure 2:**
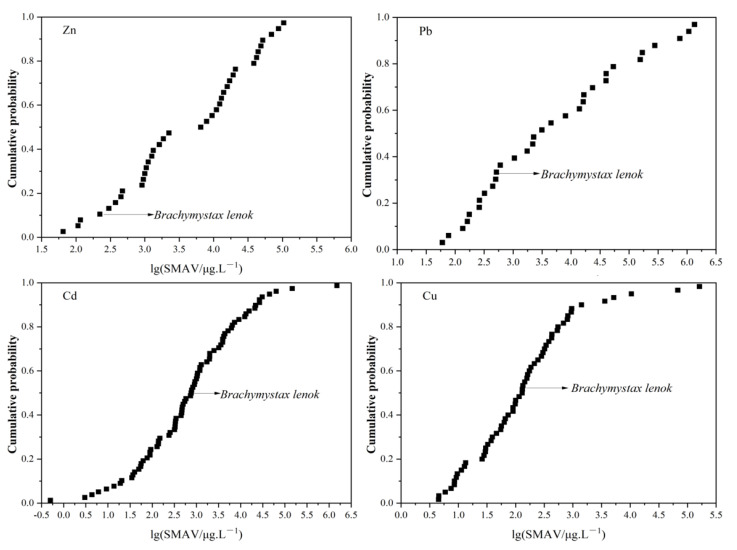
Species sensitivity distributions for the four heavy metals, across Chinese native species.

To compare the sensitivity of *B. lenok* to that of other Chinese endemics, the SMAVs of native species were selected and ranked. The sensitivities of these species were analyzed and compared (Figure 2 and Table 3). The total numbers of SMAVs obtained for native species exposed to Cu, Zn, Pb and Cd were 59, 37, 32 and 77, respectively. Within those 59, 37, 32 and 77 SMAVs, the rank of *B. lenok* was 32, 4, 11 and 38, respectively. Therefore, using the formula given above, the cumulative probabilities of Cu, Zn, Pb and Cd were calculated as 53.33%, 10.53%, 33.33% and 48.72%, respectively. As cumulative probabilities of *B. lenok* for Cu and Cd were approximately 50%, this indicated that *B. lenok* was also not sensitive to Cu and Cd in comparison to Chinese native species. The cumulative probability of *B. lenok* for Zn was less than 15%, taking into account only Chinese native species. This indicated that *B. lenok* is relatively sensitive to Zn. From the point of view of species conservation, it is more suitable to be used as a WQC development species of Zn.

### 3.4. Comparison of Sensitivity of B. lenok and Warm-Water Fish to Four Heavy Metals

It is generally believed that cold-water fish are more sensitive to pollutants than warm-water fish [29,30,33,34]. However, our results suggested that some warm-water fish were more sensitive than *B. lenok* to Cu, Pb and Cd. For example, *Megalobrama terminalis*, *Misgurnus mizolepis*, *Gambusia affinis* and *Hypophthalmichthys molitrix* were more sensitive to Cu than *B. lenok*, while *Cyprinus carpio* was more sensitive to Pb (Table 2). In addition, six warm-water fish were more sensitive to Cd than *B. lenok.* Interestingly, *B. lenok* was more sensitive to Zn than all warm-water fish for which we had data. In a previous study, the warm-water fish *Oryzias latipes* and *Cyprinus carpio* were more sensitive to nitrobenzene than the cold-water fish *Oncorhynchus mykiss* [31,35]. Similarly, the warm-water fish *Lepomis macrochirus* was more sensitive to phenanthrene than *O. mykiss* [32,36]. In addition, *Ctenopharyngodon idellus* and *Cyprinus carpio* were more sensitive to Cu than the cold-water fish *Gasterosteus aculeatus* [26,30,34,37]. Thus, our results, in conjunction with previous studies, showed that cold-water fish were not always more sensitive to pollutants than warm-water fish.

### 3.5. Development and Verification of the Model

**Figure 3:**
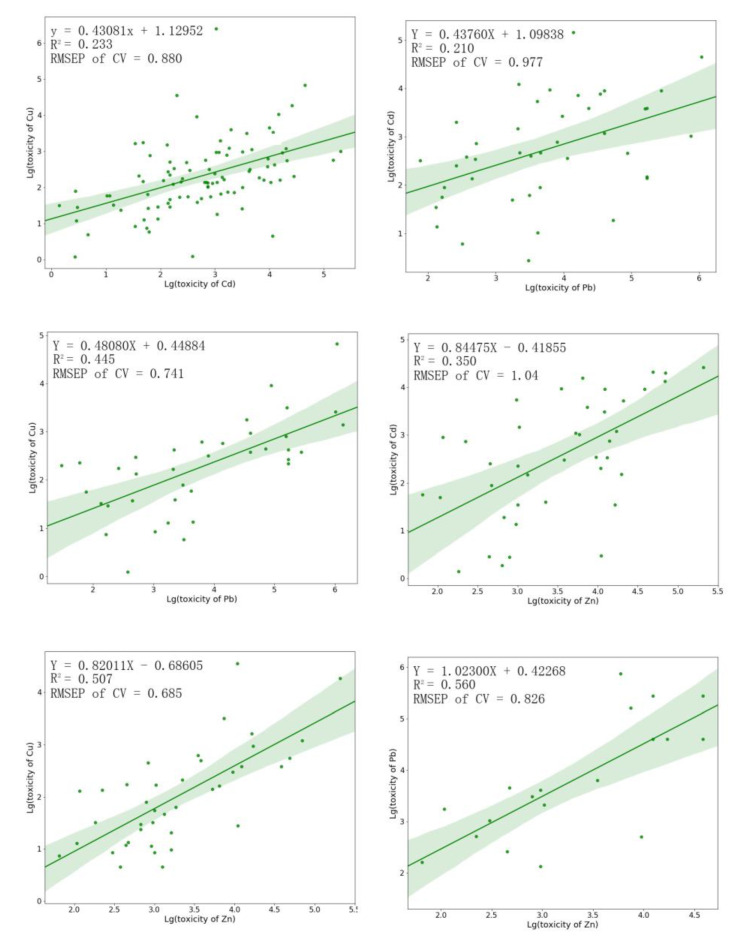
The toxicity prediction models for each pair of the four heavy metals.

Six models in total exhibited statistical significance (*p*-value < 0.05), and the square correlation coefficient (R^2^) of the models ranges from 0.210 and 0.560. The leave-one-out cross-validation result showed that the RMSEP (root-mean-square error of prediction) between the actual value and the prediction value ranges from 0.685 to 1.04 (Figure 3). The distribution of model parameters showed that 66.7% of all intercepts ranged from −0.7 and 0.7 and 100% of all slopes were within 0.4 to 1.1. This comparatively restricted variance indicates the likeness across the majority of models. The pair of Zn and Pb, and the pair of Zn and Cu showed better model performance than the other four models.

The result of external verification of the models suggested that, with the exception of the pair of Zn and Cu, and the pair of Zn and Cd, the predicted and observed values of other models all differed within five times, of which the pair of Zn and Pb performed best with a relative error of 29% (Table 4). This is an acceptable margin of error because inter-laboratory variance in acute toxicity experimental data for a certain species and chemical can reach as much as a five-fold difference for aquatic organisms [38].

This result proves that it is feasible to obtain metal biotoxicity data by using models, so that it is possible to avoid obtaining toxicity data by conducting a large number of time-consuming toxicity tests. Meanwhile, these models are simple in construction and easy to operate, making it easier for researchers and environmental managers to apply these prediction models. The alternative toxicity data provided by the simulation can be used to derive WQC and support environmental risk assessments [39,40,41].

In summary, our research leads to the following conclusions: the LC_50_ of *B. lenok* was obtained by ecotoxicological experiments, and copper was found to be the most toxic to *B. lenok*. The toxicity data of four heavy metals were screened, and the species sensitivity ranking was re-conducted based on the toxicity data of this study. The comparison of species sensitivity showed that *B. lenok* was not sensitive to copper, but it was more sensitive to Zn. We found that *B. lenok* was more suitable for the development of WQC for Zn. Cold-water fish are not necessarily more sensitive than warm-water fish, which may be due to different distribution of biota [42]. Therefore, we cannot protect warm water fish by protecting selected cold water fish. In addition, the extrapolation model we developed can predict data of one known pollutant from another, which to some extent makes up for the absence of toxicity data of Chinese native fish.

## 4. Discussion

In China, the toxicity data used for the development of WQC are partially obtained from international databases such as ECOTOX. However, the toxicity data in the database are mostly for North American species, as are cold-water fish. In addition, toxicity data are primarily obtained by studying the effects of chemical exposure on native warm-water fish. Thus, the effects of pollutants on Chinese-endemic cold-water fish are somewhat uncertain, as SSDs for these fish were not used to derive the WQC. Indeed, Chinese aquatic ecosystems and characteristic biota differ from those of North America, as do the tolerance of species and toxic effect of pollutants in different ecosystems and biota [10,42]. Therefore, it is necessary to investigate the acute effects of heavy metal exposure on a Chinese-endemic cold-water fish. Copper, zinc, lead, and cadmium are the common heavy metals contaminants in environment. To determine the sensitivity of cold-water fish to these four metals is more beneficial to the protection of aquatic organisms.

In China, fish are generally divided into eight fish regional complexes based on the area of origin [38]. *B. lenok* belongs to North mountain floristic complex [43]. A previous study identified 134 species of fish belonging to 24 families, primarily the Salmonidae, in the Great Lakes, USA [44]. Thus, the WQC derived by the US EPA were mostly based on the toxicity data of Salmonidae species [18]. However, toxicity data from other fish besides the Salmonidae are also required for the derivation of WQC. In contrast, most Chinese fish inhabit warm water, particularly species in the Cyprinidae. Thus, it was recommended that toxicity data for a cyprinid fish be used to derive WQC [11]. However, an additional, non-cyprinid fish should also be considered, preferably a cold-water species. Even though studies identifying sensitive test organisms for WQC derivation are important, these are seldom performed; even the US EPA only recommended a list of species resident in North America [18]. In previous studies, we systematically screened sensitive test organisms based on toxicity data [32,33,45,46]. These investigations of Chinese-endemic fish provide a scientific basis for WQC derivation.

It is evident that species sensitivities differ among climates. Typically, cold-water species are more sensitive than temperate and tropical species [47,48]. In the present study, our comparison of species sensitivities showed that *B. lenok* was not always more sensitive than warm-water fish. Thus, WQC protecting cold-water species might not protect species inhabiting temperate climates. However, the WQC for Cu, Zn, Pb and Cd issued by US EPA were 13, 120, 65 and 2 μg/L [47]. These concentrations were far less than 96 h-LC_50_ values of these metals for *B. lenok*. Thus, *B. lenok* would be adequately protected if the US EPA WQC were followed.

The sensitivity of a given species to chemical exposure depends on the chemical mode of action. High toxicity is associated with heavy metal bioavailability [48]. The bioavailability of heavy metals to aquatic organisms is influenced by various aquatic environmental factors, including pH, hardness, and temperature. For example, Cd toxicity is associated with water hardness and DOC (dissolved organic carbon) [49], and Zn toxicity is associated with pH [50]. Cu is influenced by a series of water chemistry parameters, including DOC, pH and temperature [51,52]. Therefore, sensitive species should be paid more attention in specific aquatic ecosystem. Further studies should focus on the sensitivity evaluation of local species to a range of aquatic contaminants.

However, some limitations should be noted. First of all, our study selected a common cold-water fish in Northeast China, while there are different species of cold-water fish in other parts of China. Secondly, the data of the model was mainly from the database, including both Chinese native species and foreign species. In further, further research on the toxicity of Chinese native species can better protect Chinese native aquatic organisms by increasing the proportion of Chinese native species in the model to optimize the prediction model.

## 5. Conclusions

The paucity of local cold-water fish data available is limitation of the development of WQC in China. In present study, acute tests of *B. lenok* exposed to four heavy metals were conducted and 96 h-LC_50_ were obtained. Comparing the toxicity values of four typical heavy metals, we found that *B. lenok* was the most sensitive to copper, followed by zinc, lead and chromium. Then, the toxicity data of the four metals were gathered from a present and a previous study, and the sensitivity of *B. lenok* was assessed. The biological ranking and cumulative probability of *B. lenok* in zinc toxicity data are lower than those of other metals, indicating that *B. lenok* is more sensitive to zinc. Thus, *B. lenok* can be considered as the test cold-water fish for derivation of Zn WQC. Our study can provide the local cold-water fish for WQC derivation and improve the applicability of WQC. The result of the prediction models of zinc and lead also demonstrates the possibility of using models to extrapolate the toxic effects of different heavy metals on the same organism.

## Figures and Tables

**Table 1:** The measured concentrations and 96 h-LC_50_ values of four heavy metals.

Pollutant	Measured Concentration (μg L^−1^)	Fitted Equation	R^2^	96 h-LC_50_ (μg L^−1^)	95% Confidence Intervals (μg L^−1^)
Cu	90, 145, 235, 365, 575	y = 2.9723x − 1.3217	0.9806	134	106–212
Zn	105, 160, 250, 370, 585	y = 4.4423x − 5.4226	0.9228	222	176–296
Pb	360, 575, 900, 1540, 2440	y = 2.8342x − 2.6829	0.9424	514	386–874
Cd	420, 610, 990, 1570, 2425	y = 3.7899x − 5.8602	0.9956	734	597–1142

**Table 2:** The sensitivity analysis of *B. lenok* to four heavy metals in comparison to other species.

Pollutants	Total Number of Species	More Sensitive Warm-Water Fish	Order	Cumulative Probability
Cu	209	*Megalobrama terminalis*	89	42.38%
		*Misgurnus mizolepis*	93	44.29%
		*Gambusia affinis*	96	45.17%
		*Hypophthalmichthys molitrix*	97	46.19%
		*Brachymystax lenok*	98	46.67%
Zn	64	*Brachymystax lenok*	6	9.23%
Pb	58	*Cyprinus carpio*	9	15.25%
		*Brachymystax lenok*	14	23.73%
Cd	245	*Oreochromis mossambicus*	31	12.60%
		*Cichlasoma facetum*	57	23.17%
		*Oryzias latipes*	64	26.02%
		*Fundulus diaphanus*	71	28.86%
		*Cyprinus carpio*	81	32.93%
		*Acrossocheilus paradoxus*	85	34.35%
		*Brachymystax lenok*	122	49.59%

**Table 3:** The sensitivity of *B. lenok* to the four heavy metals in comparison to other Chinese native species.

Pollutant	Order	Total Number of Species	Cumulative Probability
Cu	32	59	53.33%
Zn	4	37	10.53%
Pb	11	32	33.33%
Cd	38	77	48.72%

**Table 4:** External verification of the prediction models using observed data in the present study.

Surrogate Metals	Observed Value (μg/L)	Predicted Metals	Observed Value (μg/L)	Predicted Value (μg/L)	Relative Error
Zn	222	Pb	514	665	29%
		Cu	134	17	87%
		Cd	734	37	95%
Pb	514	Cu	134	57	58%
		Cd	734	193	74%
Cd	734	Cu	134	231	73%

## Data Availability

Not applicable.

## Supplementary Materials

The following supporting information can be downloaded at: https://www.mdpi.com/article/10.3390/toxics11040346/s1, Table S1: Measured DO and pH for formal test; Table S2: Concentration gradients of Cu, Zn, Pb and Cd for preliminary experiment; Table S3: The acute toxicity data of Cu; Table S4: The acute toxicity data of Zn; Table S5: The acute toxicity data of Pb; Table S6:The acute toxicity data of Cd.
