# Supplementary material for: Acute Toxicity Assessment and Prediction Models of Four Heavy Metals
Source: Toxics. 2023 Apr 6;11(4):346. doi: 10.3390/toxics11040346 (PMC10143344; doi:10.3390/toxics11040346)
Supplement: Supplementary file 1 [file toxics-11-00346-s001.zip › toxics-2178450-SI.pdf]

SUPPORTING MATERIALS

Table S1: Measured DO and pH for formal test

	Cu		Zn		Pb		Cd	
Time	Do（mg/L）	pH	Do（mg/L）	pH	Do（mg/L）	pH	Do（mg/L）	pH
0h	9.10	7.38	9.26	7.43	9.32	7.42	9.32	7.36
24 h	9.24	7.51	9.62	7.32	9.26	7.39	9.24	7.32
48 h	9.12	7.44	9.32	7.28	9.24	7.38	9.19	7.28
72 h	9.37	7.32	9.25	7.29	9.21	7.31	9.27	7.18

Table S2: Concentration gradients of Cu, Zn, Pb and Cd for preliminary experiment

Heavy metal	Control	Nominent concentration			
Cu	0	0.1	1	10	100
Zn	0	0.1	1	10	100
Pb	0	0.1	1	10	100
Cd	0	0.1	1	10	100

Table S3: The acute toxicity data of Cu

Order	Species	LC_{50}/EC_{50}(μg/L)	Source
1	*Callinectes amnicola*	0.018	ECOTOX
2	*Villorita cyprinoides*	1.214	ECOTOX
3	*Dreissena polymorpha*	1.25	ECOTOX
4	*Ceriodaphnia pulchella*	4.52	ECOTOX
5	*Clarias gariepinus*	4.54	ECOTOX
6	*Daphnia longispina*	4.56	ECOTOX
7	*Villosa fabalis*	4.6	ECOTOX
8	*Prosopium williamsoni*	5	ECOTOX
9	*Epioblasma triquetra*	5.2	ECOTOX
10	*Scapholeberis mucronata*	5.3	ECOTOX
11	*Moina macrocopa*	5.9	ECOTOX
12	*Ceriodaphnia dubia*	7.47	ECOTOX
13	*Fluminicola virens*	8	ECOTOX
14	*Daphnia galeata*	8.5	ECOTOX
15	*Ceriodaphnia reticulate*	8.56	ECOTOX
16	*Venustaconcha ellipsiformis*	8.6	ECOTOX
17	*Epioblasma torulosa*	8.7	ECOTOX

18	*Bosmina longirostris*	9.2	ECOTOX
19	*Chydorus ovalis*	9.6	ECOTOX
20	*Scapholeberis microcephala*	11.2	ECOTOX
21	*Obovaria subrotunda*	11.3	ECOTOX
22	*Simocephalus exspinosus*	11.34	ECOTOX
23	*Acroperus harpae*	11.5	ECOTOX
24	*Acantholeberis curvirostris*	11.9	ECOTOX
25	*Cottus bairdi*	12	ECOTOX
26	*Daphnia pulex*	12.82	ECOTOX
27	*Potamilus ohiensis*	13	ECOTOX
28	*Simocephalus vetulus*	13.40	ECOTOX
29	*Prochilodus scrofa*	14	ECOTOX
30	*Juga plicifera*	15	ECOTOX
31	*Ptychocheilus oregonensis*	18	ECOTOX
32	*Pomacea paludosa*	19.47	ECOTOX
33	*Acroperus elongates*	20.52	ECOTOX
34	*Lampsilis rafinesqueana*	23	ECOTOX
35	*Villosa iris*	23.24	ECOTOX
36	*Morone saxatilis*	24	ECOTOX
37	*Eurycercus lamellatus*	24.3	ECOTOX
38	*Lampsilis fasciola*	24.50	ECOTOX
39	*Leptodea leptodon*	25.26	ECOTOX
40	*Epioblasma capsaeformis*	26	ECOTOX
41	*Notemigonus crysoleucas*	26	ECOTOX
42	*Paratya compressa*	26.7	ECOTOX
43	*Hydra viridissima*	28	ECOTOX
44	*Alona quadrangularis*	28.2	ECOTOX
45	*Tropocyclops prasinus*	29	ECOTOX
46	*Gammarus pulex*	29.56	ECOTOX
47	*Cyclops viridis*	30	ECOTOX
48	*Oncorhynchus clarkia*	30	ECOTOX
49	*Oncorhynchus tshawytscha*	32	ECOTOX
50	*Daphnia magna*	32.58	ECOTOX
51	*Lampsilis siliquoidea*	34	ECOTOX
52	*Actinonaias ligamentina*	36.9	ECOTOX
53	*Daphnia carinata*	37.3	ECOTOX
54	*Duttaphrynus melanostictus*	39.1	ECOTOX
55	*Euphlyctis hexadactylus*	39.98	ECOTOX
56	*Lymnaea acuminate*	40.20	ECOTOX
57	*Disparalona rostrate*	43.3	ECOTOX
58	*Lampetra tridentate*	46	ECOTOX
59	*Lampsilis abrupta*	46.13	ECOTOX
60	*Chydorus sphaericus*	46.25	ECOTOX

61	*Potamopyrgus jenkinsi*	48	ECOTOX
62	*Biomphalaria glabrata*	49.32	ECOTOX
63	*Polypedilum nubifer*	50	ECOTOX
64	*Pleuroxus truncates*	51.6	ECOTOX
65	*Ptychobranchus fasciolaris*	54.4	ECOTOX
66	*Daphnia lumholtzi*	54.88	ECOTOX
67	*Xenocypris sp.*	55	ECOTOX
68	*Tubifex tubifex*	56.46	ECOTOX
69	*Acipenser oxyrhynchus*	60	ECOTOX
70	*Etheostoma fonticola*	60	ECOTOX
71	*Oncorhynchus kisutch*	60	ECOTOX
72	*Bellamya quadrata*	63.29	ECOTOX
73	*Diaptomus leptopus*	65	ECOTOX
74	*Spualiobarbus curriculus*	66	ECOTOX
75	*Tilapia sparrmanii*	68.1	ECOTOX
76	*Oncorhynchus gilae*	70	ECOTOX
77	*Eurytemora affinis*	71	ECOTOX
78	*Viviparus bengalensis*	73.1	ECOTOX
79	*Radix luteola*	74.03	ECOTOX
80	*Oncorhynchus mykiss*	78.6	ECOTOX
81	*Acipenser brevirostrum*	80	ECOTOX
82	*Epidalea calamita*	80	ECOTOX
83	*Hediste diversicolor*	80.6	ECOTOX
84	*Unio elongatulus*	86.34	ECOTOX
85	*Erimonax monachus*	90	ECOTOX
86	*Radix natalensis*	90	ECOTOX
87	*Barbus gonionotus*	98.34	ECOTOX
88	*Macrobrachium carcinus*	100	ECOTOX
89	*Megalobrama terminalis*	100	ECOTOX
90	*Girardia tigrina*	102.6	ECOTOX
91	*Barbus ticto*	106.4	ECOTOX
92	*Notropis mekistocholas*	110	ECOTOX
93	*Misgurnus mizolepis*	115.1	ECOTOX
94	*Bufo boreas*	120	ECOTOX
95	*Daphnia rosea*	125	ECOTOX
96	*Gambusia affinis*	129.5	ECOTOX
97	*Hypophthalmichthys molitrix*	130.8	ECOTOX
98	*Brachymystax lenok*	134	ECOTOX
99	*Rhithrogena hageni*	137	ECOTOX
100	*Moinodaphnia macleayi*	140	ECOTOX
101	*Pectinatella magnifica*	140	ECOTOX
102	*Plumatella emarginata*	140	ECOTOX
103	*Acrocheilus alutaceus*	143	ECOTOX

104	*Branchiura sowerbyi*	144.2	ECOTOX
105	*Skistodiaptomus oregonensis*	146	ECOTOX
106	*Macrobrachium lamarrei*	156.85	ECOTOX
107	*Poeciliopsis occidentalis*	160	ECOTOX
108	*Scaphirhynchus platorynchus*	160	ECOTOX
109	*Ctenopharyngodon idellus*	161.7	ECOTOX
110	*Barbus conchonius*	162.3	ECOTOX
111	*Caridina Africana*	165.1	ECOTOX
112	*Pimephales promelas*	167.5	ECOTOX
113	*Cyprinus carpio*	173.9	ECOTOX
114	*Gasterosteus aculeatus*	185.1	ECOTOX
115	*Pseudambassis ranga*	190	ECOTOX
116	*Pila globose*	193.1	ECOTOX
117	*Hyalella Azteca*	200	ECOTOX
118	*Leporinus obtusidens*	200	ECOTOX
119	*Drunella grandis*	201	ECOTOX
120	*Tilapia guineensis*	206.5	ECOTOX
121	*Gammarus lacustris*	212	ECOTOX
122	*Gila elegans*	220	ECOTOX
123	*Daphnia similis*	230	ECOTOX
124	*Erpobdella octoculata*	244.9	ECOTOX
125	*Dugesia dorotocephala*	250.5	ECOTOX
126	*Etheostoma lepidum*	260	ECOTOX
127	*Hyalella curvispina*	265	ECOTOX
128	*Xyrauchen texanus*	270	ECOTOX
129	*Physella acuta*	280	ECOTOX
130	*Megalobrama Pellegrini*	281.7	ECOTOX
131	*Danio rerio*	282.2	ECOTOX
132	*Chironomus plumosus*	300	ECOTOX
133	*Perca fluviatilis*	300	ECOTOX
134	*Etroplus maculatus*	320	ECOTOX
135	*Lumbriculus variegatus*	320	ECOTOX
136	*Planorbella trivolvis*	320	ECOTOX
137	*Rasbora daniconius*	323.7	ECOTOX
138	*Etheostoma flabellare*	330	ECOTOX
139	*Ceriodaphnia rigaudi*	340	ECOTOX
140	*Carassius auratus*	380.1	ECOTOX
141	*Gammarus fasciatus*	390	ECOTOX
142	*Chironomus tentans*	425.7	ECOTOX
143	*Hoplobatrachus tigerinus*	427.5	ECOTOX
144	*Bufo gargarizans*	429.5	ECOTOX
145	*Ptychocheilus Lucius*	430	ECOTOX
146	*Elimia livescens*	440	ECOTOX

147	*Macrobrachium rosenbergii*	453	ECOTOX
148	*Planorbis planorbis*	460	ECOTOX
149	*Echinogammarus berilloni*	467.2	ECOTOX
150	*Etheostoma nigrum*	483	ECOTOX
151	*Culicoides furens*	500	ECOTOX
152	*Lophopodella carteri*	510	ECOTOX
153	*Spinibarbus sinensis*	534	ECOTOX
154	*Rana limnocharis*	548.6	ECOTOX
155	*Cherax destructor*	576.4	ECOTOX
156	*Aphelenchus avenae*	576.8	ECOTOX
157	*Rutilus rutilus*	590	ECOTOX
158	*Macrobrachium dayanum*	603.4	ECOTOX
159	*Peprilus triacanthus*	612.1	ECOTOX
160	*Lepidocephalichthys guntea*	617.2	ECOTOX
161	*Lepomis macrochirus*	620	ECOTOX
162	*Cyprinodon variegatus*	630	ECOTOX
163	*Ictalurus furcatus*	686	ECOTOX
164	*Biomphalaria alexandrina*	709.9	ECOTOX
165	*Chironomus decorus*	739	ECOTOX
166	*Anodontites trapesialis*	762.1	ECOTOX
167	*Lymnaea stagnalis*	800	ECOTOX
168	*Dugesia japonica*	816.1	ECOTOX
169	*Cephalobus persegnis*	920.3	ECOTOX
170	*Cypris subglobosa*	935.2	ECOTOX
171	*Diaphanosoma brachyurum*	950	ECOTOX
172	*Xenopus laevis*	965.2	ECOTOX
173	*Pelophylax perezi*	970	ECOTOX
174	*Channa marulius*	977.6	ECOTOX
175	*Chironomus riparius*	1000	ECOTOX
176	*Ictalurus punctatus*	1142	ECOTOX
177	*Mystus bleekeri*	1146	ECOTOX
178	*Oreochromis niloticus*	1207	ECOTOX
179	*Lepomis gibbosus*	1240	ECOTOX
180	*Cyprinodon bovinus*	1300	ECOTOX
181	*Colisa fasciata*	1400	ECOTOX
182	*Poecilia reticulate*	1409	ECOTOX
183	*Cirrhinus mrigala*	1500	ECOTOX
184	*Oreochromis mossambicus*	1628	ECOTOX
185	*Macrobrachium hendersodayanus*	1750	ECOTOX
186	*Crangonyx pseudogracilis*	1774	ECOTOX
187	*Catostomus commersoni*	1984	ECOTOX
188	*Corbicula manilensis*	2600	ECOTOX

189	*Helisoma duryi*	2800	ECOTOX
190	*Dugesia tigrina*	3200	ECOTOX
191	*Esomus danricus*	3287	ECOTOX
192	*Barilius vagra*	3400	ECOTOX
193	*Lepomis cyanellus*	3400	ECOTOX
194	*Melanoides tuberculate*	3600	ECOTOX
195	*Clarias lazera*	3814	ECOTOX
196	*Rhinella arenarum*	4000	ECOTOX
197	*Lamellidens marginalis*	4466	ECOTOX
198	*Microhyla ornate*	5162	ECOTOX
199	*Asellus aquaticus*	9210	ECOTOX
200	*Heteropneustes fossilis*	10490	ECOTOX
201	*Tympanotonus fuscatus*	11097	ECOTOX
202	*Mystus vittatus*	18620	ECOTOX
203	*Oziotelphusa senex*	24165	ECOTOX
204	*Asellus intermedius*	32000	ECOTOX
205	*Labeo rohita*	35707	ECOTOX
206	*Sinopotamon honanense*	67654	ECOTOX
207	*Procambarus clarkia*	162000	ECOTOX
208	*Spiralothelphusa hydrodroma*	254680	ECOTOX
209	*Lepidocephalichthys thermalis*	2500000	ECOTOX

Table S4: The acute toxicity data of Zn

Order	Species	LC_{50}/EC_{50}(μg/L)	Source
1	*Anodonta cygnea*	56.2	ECOTOX
2	*Ceriodaphnia dubia*	65	ECOTOX
3	*Daphnia pulex*	107	ECOTOX
4	*Gambusia affinis*	116	ECOTOX
5	*Oncorhynchus tshawytscha*	182	ECOTOX
6	*Brachymystax lenok*	222	ECOTOX
7	*Ceriodaphnia reticulate*	300	ECOTOX
8	*Daphnia longispina*	375	ECOTOX
9	*Cottus bairdi*	439	ECOTOX
10	*Cyprinus carpio*	450	ECOTOX
11	*Simocephalus vetulus*	473	ECOTOX
12	*Salmo trutta*	640	ECOTOX
13	*Morone saxatilis*	670	ECOTOX
14	*Oncorhynchus clarkia*	670	ECOTOX
15	*Oncorhynchus mykiss*	790	ECOTOX
16	*Macrobrachium rosenbergii*	832	ECOTOX
17	*Simocephalus exspinosus*	911	ECOTOX
18	*Daphnia magna*	952	ECOTOX

19	*Salvelinus fontinalis*	960	ECOTOX
20	*Physastra gibbosa*	1000	ECOTOX
21	*Daphnia galeata*	1001	ECOTOX
22	*Daphnia lumholtzi*	1001	ECOTOX
23	*Pimephales promelas*	1039	ECOTOX
24	*Misgurouaanguillicaudatus*	1050	ECOTOX
25	*Acipensers chrenckii Brandt*	1119	ECOTOX
26	*Ceriodaphnia pulchella*	1263	ECOTOX
27	*Chydorus sphaericus*	1326	ECOTOX
28	*Acroperus elongates*	1614	ECOTOX
29	*Chydorus ovalis*	1627	ECOTOX
30	*Bellamya quadrata*	1862	ECOTOX
31	*Gammarus lacustris*	2240	ECOTOX
32	*Lepomis macrochirus*	3500	ECOTOX
33	*Culicoides furens*	3800	ECOTOX
34	*Plumatella emarginata*	5300	ECOTOX
35	*Procambarus clarkii*	5897	ECOTOX
36	*Ctenopharyngodon idellus*	6494	ECOTOX
37	*Dugesia tigrina*	7400	ECOTOX
38	*Eriocheir sinensis*	7908	ECOTOX
39	*Chironomus plumosus*	9500	ECOTOX
40	*Labeo rohita*	10910	ECOTOX
41	*Hydra viridissima*	11000	ECOTOX
42	*Morone americana*	12060	ECOTOX
43	*Carassius auratus*	12259	ECOTOX
44	*Hydra vulgaris*	13000	ECOTOX
45	*Hydra oligactis*	14000	ECOTOX
46	*Pseudorasbora parva*	15794	ECOTOX
47	*Oreochromis mossambicus*	16500	ECOTOX
48	*Anguilla rostrata*	17072	ECOTOX
49	*Cypris subglobosa*	17095	ECOTOX
50	*Rhodeus ocellatus*	19225	ECOTOX
51	*Fundulus diaphanus*	19791	ECOTOX
52	*Tanichthys albonubes*	20750	ECOTOX
53	*Lepomis gibbosus*	20981	ECOTOX
54	*Carassius auratus*	38356	ECOTOX
55	*Cipangopaludina chinensis*	42173	ECOTOX
56	*Carassius auratus*	44341	ECOTOX
57	*Rana limnocharis*	48698	ECOTOX
58	*Pseudogobius masago*	51761	ECOTOX
59	*Channa punctata*	69282	ECOTOX
60	*Oreochromis niloticus*	70004	ECOTOX
61	*Rana catesbeiana*	87750	ECOTOX

62	*Cryprinus carpiod*	104504	ECOTOX
63	*Cichlasoma managuense*	108153	ECOTOX
64	*Mystus vittatus*	209000	ECOTOX

Table S5: The acute toxicity data of Pb

Order	Species	LC_{50}/EC_{50}(μg/L)	Source
1	*Hyalella Azteca*	30	ECOTOX
2	*Daphnia similis*	60	ECOTOX
3	*Tubifex tubifex*	77	ECOTOX
4	*Gammarus pseudolimnaeus*	132	ECOTOX
5	*Daphnia magna*	135	ECOTOX
6	*Ceriodaphnia dubia*	162	ECOTOX
7	*Gammarus pulex*	175	ECOTOX
8	*Caenorhabditis elegans*	260	ECOTOX
9	*Cyprinus carpio*	262	ECOTOX
10	*Thymallus arcticus*	320	ECOTOX
11	*Dreissena polymorpha*	370	ECOTOX
12	*Daphnia carinata*	444	ECOTOX
13	*Chironomus plumosus*	500	ECOTOX
14	*Brachymystax lenok*	514	ECOTOX
15	*Daphnia hyaline*	600	ECOTOX
16	*Baetis tricaudatus*	664	ECOTOX
17	*Ceriodaphnia reticulate*	1050	ECOTOX
18	*Daphnia pulex*	1745	ECOTOX
19	*Pimephales promelas*	2100	ECOTOX
20	*Chironomus tentans*	2178	ECOTOX
21	*Micropterus dolomieu*	2200	ECOTOX
22	*Duttaphrynus melanostictus*	2260	ECOTOX
23	*Oncorhynchus mykiss*	3059	ECOTOX
24	*Moina macrocopa*	3133	ECOTOX
25	*Orconectes limosus*	3300	ECOTOX
26	*Chironomus dilutes*	3323	ECOTOX
27	*Salvelinus fontinalis*	4100	ECOTOX
28	*Oncorhynchus kisutch*	4180	ECOTOX
29	*Simocephalus vetulus*	4500	ECOTOX
30	*Mesocyclops hyalinus*	4561	ECOTOX
31	*Lepomis macrochirus*	6300	ECOTOX
32	*Lumbriculus variegatus*	8000	ECOTOX
33	*Amnicola limosa*	9500	ECOTOX
34	*Pisidium compressum*	11400	ECOTOX
35	*Aphelenchus avenae*	13885	ECOTOX
36	*Lymnaea emarginata*	14000	ECOTOX
37	*Rhodeus sinensis*	16174	ECOTOX

38	*Channa punctate*	16600	ECOTOX
39	*Limnodrilus hoffmeisteri*	23441	ECOTOX
40	*Crangonyx pseudogracilis*	34769	ECOTOX
41	*Carassius auratus*	40000	ECOTOX
42	*Cypris subglobosa*	40190	ECOTOX
43	*Caridina nilotica*	53500	ECOTOX
44	*Elimia livescens*	71000	ECOTOX
45	*Asellus aquaticus*	87704	ECOTOX
46	*Dugesia japonica*	155503	ECOTOX
47	*Dugesia tigrina*	160000	ECOTOX
48	*Eriocheir sinensis*	169262	ECOTOX
49	*Gila elegans*	170000	ECOTOX
50	*Ptychocheilus Lucius*	170000	ECOTOX
51	*Xyrauchen texanus*	170000	ECOTOX
52	*Tilapia hornorum*	202000	ECOTOX
53	*Tanytarsus dissimilis*	224000	ECOTOX
54	*Carassius auratus*	277546	ECOTOX
55	*Procambarus clarkii*	751570	ECOTOX
56	*Corbicula manilensis*	1023320	ECOTOX
57	*Sinopotamon honanense*	1072256	ECOTOX
58	*Poecilia reticulate*	1346243	ECOTOX

Table S6: The acute toxicity data of Cd

Order	Species	LC_{50}/EC_{50}(μg/L)	Source
1	*Acanthocyclops viridis*	0.5	ECOTOX
2	*Megacyclops viridis*	0.5	ECOTOX
3	*Salvelinus confluentus*	0.9	ECOTOX
4	*Oncorhynchus tshawytscha*	1.4	ECOTOX
5	*Salmo trutta*	1.87	ECOTOX
6	*Villorita cyprinoides*	2.7	ECOTOX
7	*Oncorhynchus mykiss*	2.78	ECOTOX
8	*Cottus bairdi*	2.9	ECOTOX
9	*Hydra viridissima*	3	ECOTOX
10	*Moina irrasa*	4.34	ECOTOX
11	*Prosopium williamsoni*	4.7	ECOTOX
12	*Thymallus arcticus*	6.1	ECOTOX
13	*Pseudosida ramosa*	8	ECOTOX
14	*Simocephalus serrulatus*	9.26	ECOTOX
15	*Oncorhynchus kisutch*	10.4	ECOTOX
16	*Lampsilis teres*	11	ECOTOX
17	*Etheostoma fonticola*	11.77	ECOTOX
18	*Daphnia magna*	13.7	ECOTOX
19	*Anodonta cygnea*	14.9	ECOTOX

20	*Daphnia ambigua*	16.47	ECOTOX
21	*Caridina nilotica*	19	ECOTOX
22	*Morone saxatilis*	19	ECOTOX
23	*Hyalella azteca*	20	ECOTOX
24	*Macrobranchium nipponense*	20.4	ECOTOX
25	*Macrobrachium rude*	21.04	ECOTOX
26	*Anodonta imbecillis*	22.6	ECOTOX
27	*Nephelopsis obscura*	22.97	ECOTOX
28	*Villosa vibex*	30	ECOTOX
29	*Gammarus pseudolimnaeus*	34.59	ECOTOX
30	*Daphnia galeata*	34.64	ECOTOX
31	*Oreochromis mossambicus*	34.77	ECOTOX
32	*Brachionus calyciflorus*	36.59	ECOTOX
33	*Lampsilis stramine*	38	ECOTOX
34	*Gammarus lacustris*	40.1	ECOTOX
35	*Echinogammarus meridionalis*	44.15	ECOTOX
36	*Actinonaias pectorosa*	46	ECOTOX
37	*Macrobrachium hendersodayanus*	47.72	ECOTOX
38	*Skistodiaptomus oregonensis*	48	ECOTOX
39	*Daphnia pulex*	49.93	ECOTOX
40	*Atyaephyra desmarestii*	51.82	ECOTOX
41	*Daphnia hyalina*	55	ECOTOX
42	*Ceriodaphnia dubia*	56.3	ECOTOX
43	*Diaptomus leptopus*	59	ECOTOX
44	*Paratya compressa*	59.2	ECOTOX
45	*Paratya australiensis*	60	ECOTOX
46	*Moina macrocopa*	62.5	ECOTOX
47	*Anodontites trapesialis*	64	ECOTOX
48	*Gammarus fossarum*	70	ECOTOX
49	*Moina dubia*	77	ECOTOX
50	*Ptychocheilus lucius*	78	ECOTOX
51	*Utterbackia imbecillis*	82	ECOTOX
52	*Simocephalus vetulus*	89.3	ECOTOX
53	*Gammarus pulex*	89.4	ECOTOX
54	*Daphnia cucullata*	90.1	ECOTOX
55	*Aplexa hypnorum*	93	ECOTOX
56	*Heliodiaptomus viduus*	94.8	ECOTOX
57	*Cichlasoma facetum*	95.39	ECOTOX
58	*Macrobrachium lamarrei*	114.1	ECOTOX
59	*Ceriodaphnia reticulata*	129	ECOTOX
60	*Cirrhinus mrigala*	132	ECOTOX
61	*Spirostomum ambiguum*	137.2	ECOTOX

62	*Daphnia carinata*	137.9	ECOTOX
63	*Xyrauchen texanus*	139	ECOTOX
64	*Oryzias latipes*	139.1	ECOTOX
65	*Echinisca triserialis*	141.4	ECOTOX
66	*Gila elegans*	148	ECOTOX
67	*Chydorus sphaericus*	149	ECOTOX
68	*Tropocyclops prasinus*	149	ECOTOX
69	*Lirceus alabamae*	150	ECOTOX
70	*Lophopodella carteri*	150	ECOTOX
71	*Fundulus diaphanus*	152	ECOTOX
72	*Daphnia rosea*	171	ECOTOX
73	*Ceriodaphnia rigaudi*	178.8	ECOTOX
74	*Labeo rohita*	201	ECOTOX
75	*Pristina leidyi*	214.6	ECOTOX
76	*Austropotamobius pallipes*	220	ECOTOX
77	*Philodina acuticornis*	223.6	ECOTOX
78	*Daphnia lumholtzi*	226.5	ECOTOX
79	*Branchiura sowerbyi*	240	ECOTOX
80	*Micropterus salmoides*	244.1	ECOTOX
81	*Cyprinus carpio*	253.6	ECOTOX
82	*Culicoides furens*	300	ECOTOX
83	*Quistadrilus multisetosus*	320	ECOTOX
84	*Tubifex tubifex*	320	ECOTOX
85	*Acrossocheilus paradoxus*	328.8	ECOTOX
86	*Baetis rhodani*	331.6	ECOTOX
87	*Hydra vulgaris*	336.6	ECOTOX
88	*Chironomus plumosus*	346.4	ECOTOX
89	*Peloscolex ferox*	350	ECOTOX
90	*Ranatra elongata*	355.1	ECOTOX
91	*Etheostoma spectabile*	355.9	ECOTOX
92	*Pisidium compressum*	360	ECOTOX
93	*Physa fontinalis*	379.4	ECOTOX
94	*Dreissena polymorpha*	388	ECOTOX
95	*Orconectes limosus*	400	ECOTOX
96	*Nitocra spinipes*	430	ECOTOX
97	*Paraleptophlebia praepedita*	449	ECOTOX
98	*Spirosperma nikolskyi*	450	ECOTOX
99	*Asellus aquaticus*	464.7	ECOTOX
100	*Mesocyclops hyalinus*	466.3	ECOTOX
101	*Ambystoma gracile*	468.4	ECOTOX
102	*Duttaphrynus melanostictus*	471.7	ECOTOX
103	*Echinogammarus echinosetosus*	480	ECOTOX
104	*Glossiphonia complanata*	480	ECOTOX

105	*Vorticella microstoma*	490	ECOTOX
106	*Cyclops varicans*	493	ECOTOX
107	*Aedes aegypti*	500	ECOTOX
108	*Callinectes sapidus*	500	ECOTOX
109	*Physa gyrina*	531.8	ECOTOX
110	*Alona affinis*	546	ECOTOX
111	*Eudiaptomus padanus*	550	ECOTOX
112	*Stylodrilus heringianus*	550	ECOTOX
113	*Biomphalaria glabrata*	565.9	ECOTOX
114	*Macrobrachium kistnensis*	569.6	ECOTOX
115	*Daphnia obtusa*	580	ECOTOX
116	*Ambystoma mexicanum*	620	ECOTOX
117	*Rhyacodrilus montanus*	630	ECOTOX
118	*Caridina rajadhari*	630.7	ECOTOX
119	*Moinodaphnia macleayi*	650	ECOTOX
120	*Cherax destructor*	672.1	ECOTOX
121	*Pectinatella magnifica*	700	ECOTOX
122	*Brachymystax lenok*	734	ECOTOX
123	*Girardia tigrina*	740	ECOTOX
124	*Barbus ticto*	740	ECOTOX
125	*Hydra oligactis*	761	ECOTOX
126	*Lumbriculus variegatus*	780	ECOTOX
127	*Xenocypris sp.*	830	ECOTOX
128	*Atalophlebia australis*	840	ECOTOX
129	*Gambusia affinis*	900	ECOTOX
130	*Gammarus italicus*	910	ECOTOX
131	*Cloeon dipterum*	926.3	ECOTOX
132	*Anguilla rostrata*	949.7	ECOTOX
133	*Erpobdella octoculata*	1000	ECOTOX
134	*Procambarus clarkii*	1040	ECOTOX
135	*Lepidocephalichthys thermalis*	1045.8	ECOTOX
136	*Diaphanosoma brachyurum*	1060	ECOTOX
137	*Plumatella emarginata*	1090	ECOTOX
138	*Ptychocheilus oregonensis*	1092	ECOTOX
139	*Echinogammarus tibaldii*	1100	ECOTOX
140	*Cypris subglobosa*	1194.5	ECOTOX
141	*Aeolosoma headleyi*	1200	ECOTOX
142	*Catostomus commersoni*	1257.3	ECOTOX
143	*Spualiobarbus curriculus*	1290	ECOTOX
144	*Fundulus heteroclitus*	1300	ECOTOX
145	*Pseudambassis ranga*	1350	ECOTOX
146	*Pimephales promelas*	1476.2	ECOTOX
147	*Barytelphusa guerini*	1548.1	ECOTOX

148	*Chasmagnathus granulata*	1580	ECOTOX
149	*Lymnaea stagnalis*	1650	ECOTOX
150	*Lymnaea acuminata*	1653.8	ECOTOX
151	*Paratelphusa hydrodromus*	1686.5	ECOTOX
152	*Radix luteola*	1722	ECOTOX
153	*Lepomis gibbosus*	1816	ECOTOX
154	*Rhinella arenarum*	1954	ECOTOX
155	*Microhyla ornata*	1972	ECOTOX
156	*Caenorhabditis elegans*	2000	ECOTOX
157	*Ephemerella subvaria*	2000	ECOTOX
158	*Viviparus bengalensis*	2259	ECOTOX
159	*Jordanella floridae*	2500	ECOTOX
160	*Radix plicatula*	2500	ECOTOX
161	*Amnicola limosa*	2710	ECOTOX
162	*Nuria danrica*	2962	ECOTOX
163	*Morone americana*	3039	ECOTOX
164	*Notemigonus crysoleucas*	3150	ECOTOX
165	*Megalobrama terminalis*	3200	ECOTOX
166	*Xenopus laevis*	3200	ECOTOX
167	*Spinibarbus denticulatus*	3572	ECOTOX
168	*Cyclops abyssorum*	3800	ECOTOX
169	*Dugesia tigrina*	3853	ECOTOX
170	*Limnodrilus hoffmeisteri*	3919	ECOTOX
171	*Eriocheir sinensis*	3928	ECOTOX
172	*Poecilia reticulata*	4154	ECOTOX
173	*Danio rerio*	4200	ECOTOX
174	*Diacypris compacta*	4340	ECOTOX
175	*Diaptomus forbesi*	4347	ECOTOX
176	*Culex pipiens*	4400	ECOTOX
177	*Etroplus maculatus*	4400	ECOTOX
178	*Ictalurus punctatus*	4697	ECOTOX
179	*Crassostrea rhizophorae*	5000	ECOTOX
180	*Tanichthys albonubes*	5188	ECOTOX
181	*Garra mullya*	5400	ECOTOX
182	*Salvelinus fontinalis*	5473	ECOTOX
183	*Niphargus aquilex*	5809	ECOTOX
184	*Stenocypris malcolmsoni*	5970	ECOTOX
185	*Orconectes virilis*	6100	ECOTOX
186	*Megalobrama pellegrini*	6187	ECOTOX
187	*Hexagenia rigida*	6200	ECOTOX
188	*Penaeus duorarum*	6491	ECOTOX
189	*Gasterosteus aculeatus*	6500	ECOTOX
190	*Cyprinella lutrensis*	6620	ECOTOX

191	*Rhodeus sinensis*	7283	ECOTOX
192	*Crangonyx pseudogracilis*	7669	ECOTOX
193	*Barbus conchonius*	7800	ECOTOX
194	*Parreysia favidens*	8417	ECOTOX
195	*Carassius auratus*	9124	ECOTOX
196	*Lepomis macrochirus*	9393	ECOTOX
197	*Panagrellus silusiae*	9398	ECOTOX
198	*Lamellidens marginalis*	10000	ECOTOX
199	*Orconectes immunis*	10200	ECOTOX
200	*Rhithrogena hageni*	10500	ECOTOX
201	*Clarias gariepinus*	11410	ECOTOX
202	*Lepomis cyanellus*	11520	ECOTOX
203	*Chironomus tentans*	12393	ECOTOX
204	*Channa punctata*	13203	ECOTOX
205	*Perca flavescens*	13253	ECOTOX
206	*Heteropneustes fossilis*	14600	ECOTOX
207	*Ephemerella ignita*	14696	ECOTOX
208	*Ctenopharyngodon idellus*	15540	ECOTOX
209	*Rana luteiventris*	16325	ECOTOX
210	*Cephalobus persegnis*	17164	ECOTOX
211	*Pteronarcella badia*	18000	ECOTOX
212	*Oreochromis niloticus*	19919	ECOTOX
213	*Rana limnocharis*	20984	ECOTOX
214	*Dorylaimus stagnalis*	21782	ECOTOX
215	*Plectus communis*	21830	ECOTOX
216	*Dendrocoelum lacteum*	23220	ECOTOX
217	*Nemoura cinerea*	23298	ECOTOX
218	*Ischnura heterosticta*	23300	ECOTOX
219	*Danio malabaricus*	25000	ECOTOX
220	*Mystus vittatus*	25820	ECOTOX
221	*Corixa punctata*	26626	ECOTOX
222	*Dugesia lugubris*	26626	ECOTOX
223	*Ischnura elegans*	26626	ECOTOX
224	*Tobrilus gracilis*	26670	ECOTOX
225	*Drunella grandis*	28000	ECOTOX
226	*Aporcelaimellus obtusicaudatus*	30711	ECOTOX
227	*Polycelis felina*	32310	ECOTOX
228	*Leuctra inermis*	41353	ECOTOX
229	*Barbus arulius*	42044	ECOTOX
230	*Sinopotamon honanense*	45032	ECOTOX
231	*Notopterus notopterus*	63489	ECOTOX
232	*Pelophylax ridibundus*	71800	ECOTOX
233	*Polycelis tenuis*	86023	ECOTOX

234	*Acrobeloides buetschlii*	121685	ECOTOX
235	*Aphelenchus avenae*	146400	ECOTOX
236	*Tylenchus elegans*	146400	ECOTOX
237	*Chironomus riparius*	200000	ECOTOX
238	*Hydropsyche angustipennis*	200000	ECOTOX
239	*Rhyacophila dorsalis*	400000	ECOTOX
240	*Isoperla grammatica*	420000	ECOTOX
241	*Dinocras cephalotes*	916515	ECOTOX
242	*Enallagma cyathigerum*	1372953	ECOTOX
243	*Calopteryx splendens*	1500000	ECOTOX
244	*Sigara dorsalis*	3532704	ECOTOX
245	*Aphelocheirus aestivalis*	12000000	ECOTOX

As to the source of the data, they are all from ECOTOX database and there are more than six hundreds articles mentioned. And the details of references were not shown in Table S3- Table S6.
